# Self-Care Behaviors among Thai Primigravida Teenagers

**DOI:** 10.5539/gjhs.v4n3p139

**Published:** 2012-05-01

**Authors:** Suphawadee Panthumas, Wirin Kittipichai, Supachai Pitikultang, Kanittha Chamroonsawasdi

**Affiliations:** 1 Department of Family Health, Faculty of Public Health, Mahidol University, Bangkok, Thailand

**Keywords:** teenager, primigravida, self-care behavior, self-esteem, perceived self-efficacy

## Abstract

The purpose of this study was to investigate predictive factors of the self-care behaviors among Thai teenagers with primigravida. The samples of 206 primigravida teenagers attending ANC clinics of six hospitals in the North-Eastern region of Thailand were included. Data collection was done through self administered-questionnaire. Scales of the questionnaire had reliability coefficients ranging from 0.72 – 0.92. The data were analyzed by using descriptive and inferential statistics. The results revealed that the percentage-mean score of overall self-care behavior was 76.91. The percentage-mean scores of self-care behaviors in specific trimester were found that the score in the second trimester was lower than the scores in the first and third trimesters (57.58, 60.45, and 64.65, respectively). Factors associated with overall self-care behavior were perceived self-efficacy, perceived social support from family, knowledge on self-care during pregnancy, accessibility to health services, self-esteem and age (r = 0.47, 0.34, 0.28, 0.24, 0.19, and 0.15, respectively). Perceived self-efficacy and knowledge on self-care during pregnancy were the two considerable predictors accounted for 25% of the variance in the self-care behaviors of Thai teenagers with primigravida.

## 1. Introduction

According to the teenage pregnancy survey by WHO in 2010, the global average was 65 pregnancies per 1,000 females while it was 56 in Asia and 70 in Thailand. An increased rate of teenage pregnancy leads to an increased risk of adverse perinatal outcome. The babies of pregnant teenagers are more likely to be born prematurely or the pregnancy results in stillbirth (World Health Organization, 2010). Infants born from teenage pregnancy had higher rates of extreme prematurity and higher rates of the newborn intensive care unit (NICU) admission than those born to pregnant adults (Shrim, Ates, Mallozzi, et al, 2011). Perinatal, neonatal mortality, low birth weight and congenital anomalies are also common (Shrim, Ates, Mallozzi, et al, 2011). Most Brazilian pregnant teenagers usually have their first ANC at more than 12 weeks of gestation and less than six visits in total (Cesar, Mendoza-Sassi, Gonzalez-Chica et al, 2011). With higher mental health problems such as anxiety or depressive symptoms, prior use of tobacco, dropping out of school, suicide of a social acquaintance, preventive mental health care is needed to help pregnant teenagers (Freitas, Cais, Stefanello, et al, 2008). Furthermore, the studies in rural areas found that pregnant teenagers had stress and needs which in turn affected their health-related behaviors from a public health nursing perspective. (Chen, James, Hsu, et al, 2005). A study in Swedish mothers found that teenage pregnancy had higher risky health behaviors with lower perception of social support, lower self-esteem, and more depressive symptoms than adult mothers. (Wahn & Nissen, 2011). Seemingly, in New Zealand, pregnant teenagers have been associated with negative health outcomes, health professionals also employed a motherhood discourse that attributes certain behaviors to good mothers. (Breheny & Stephens, 2007). Studies in developing countries also showed that the first-born children of pregnant teenagers are the most vulnerable to infant mortality and poor child health outcomes (Finlay, Ozaltin & Canning, 2011). At an institutional level, it is attributed to the lack of public policies and consequently, of services addressed to and adequate to health specificities within rural settlements. (Soares & Lopes, 2011). Although public health initiatives have been successful in decreasing rates of pregnant teenagers, these remain high risk pregnancies that may benefit from centers capable of ensuring adequate prenatal care. (Malabarey, Balayla, Klam, et al, 2011).

In Thailand, Chayathab (2006) found that pregnant adolescents lived in the area of Bangkok metropolitan had more instances of anemia (42.5%) and less body weight gain (40%). Data from a province in the North-Eastern region of Thailand found that the teenage pregnancy rate was 19.75% and common health problems were anemia (9.45%), antenatal care later than 12 weeks (46.63%), less than four antenatal visits (16.68%), perinatal mortality (3.58%) and low birth weight babies (8.92%) (Rojsutapong, 2011). Evidence suggests that the optimum self-care behaviors or lifestyle during pregnancy will help the pregnant teenagers and the fetus to maintain good health. (Viboonwatthanakitt, Pancharean & Tipalonkot, 2007).

The most pregnant teenagers are unplanned pregnancy and unsuitable behaviors during pregnancy. They had higher risky complications during pregnancy than pregnant adults. The self-care behaviors are important and necessary for pregnancy among teenagers. If teenagers are proper behaviors during pregnancy both they and their babies will get healthy as well. The self-care in the meaning of Orem is the practice of activities by human to maintain their lives, health and well-being. Orem’s self-care deficit theory consist of the structure of self-care behavior has four related-categories namely the self-care, the self-care agency, the therapeutic self-care demand and the general of basic conditioning factors (Orem, 2001). This present, the self-care behaviors of primigravida teenager means activities and daily life habits that would prevent complication of pregnancy as well as maintain good health conditions all through the pregnancy.

Previous studies showed the factors associated with self-care behaviors of pregnant teenagers include: household economic status (Schwartz, Vieira & Geib, 2011), accessibility to health services (Rachel, Simon & Elizabeth, et al, 2011), perceived social support (Lertsakornsiri, 2009; Schwartz, Vieira & Geib, 2011; Wahn & Nissen, 2008), self-esteem (Wahn & Nissen, 2008) perceived self-efficacy (Callaghan, 2006; Lertsakornsiri, 2009) and self-care knowledge (Wise & Arcamone, 2011). There was also a study on perceived self-efficacy theory of Bandura and self-care theory of Orem by Callaghan in 2006 wherein the results found that the support system, adequate income, adequate living conditions, routine practice of religion, and reported medical problems or disabilities were significant factors contributing towards healthy behaviors.

This study focused on the self-care behavior of Thai teenagers with primigravida because self-care is necessary and of the utmost importance during pregnancy. The Orem’s self-care deficit theory in 2001 was applied in the conceptual framework and general, basic conditioning factors and abilities for self-care were included. Basic conditioning factors consisted of (1) personal factors: age, education level, marital status, work status, economic status, current gestational age (current GA), GA at first ANC, plan for the current pregnancy, plan for self child rearing of their babies and accessibility to health services (AHS) and (2) family factors: family type and perceived social support from family (SSF). Abilities for self-care consisted of three variables namely self-esteem (SE), knowledge on self-care during pregnancy (KN) and perceived self-efficacy of self-care (SEF). Finally, the finding of the study may provide useful information for the health personnel to encourage appropriate self-care behaviors in pregnant teenagers leading to less complications, quality pregnancy and quality of life.

## 2. Objective of the Research

The objective was to investigate predictive factors of the self-care behaviors among Thai teenagers with primigravida in a selected area of Thailand.

## 3. Methods

### 3.1 Research Design and Sample

This study was a cross-sectional research conducted among 206 Thai teenagers with primigravida who attended the antenatal care clinics (ANC) of the six hospitals, in a selected area of the North-Eastern region of Thailand. The participants were included using the stratified sampling method. Among strata, hospital sizes were categorized as big, medium, and small. Inclusion criteria were the first pregnant teenagers aged 13-19 years with no complications during the pregnancy such as anemia, placenta previa, bleeding per vagina, pregnancy induced hypertension, and diabetes, literacy, and informed consent signed by themselves or by a parent in cases where the participant was younger than 18 years of age. Data was collected between January and February 2012.

### 3.2 Research Instruments

The research instrument was the self-administered questionnaire which consisted of 7 parts. *Part 1 Personal information*: family type, age, education level, marital status, work status, economic status, current GA, GA at first ANC, plan for the current pregnancy, plan for self child rearing of their babies and age, education level, work status and 3 items of their husbands. *Part 2 Accessibility to health service*: 5 items were conducted that focusing on getting convenience and satisfaction of the all service in hospital including health providers. *Part 3 Perceived social support from family*: 10 items were conducted from the social support concept of House (1985). *Part 4 Self-esteem scale*: 10 items were adapted from Rosenberg’s self-esteem scale (Rosenberg, 1965). *Part 5 Perceived self-efficacy of self-care*: scale was utilized the perceived self-efficacy theory of Bandura (1997) by developing 10 items. In support on part 2 to 5, each item on a 5-point Likert scale ranging from 1 (completely disagree) to 5 (completely agree). *Part 6 Knowledge on self-care during pregnancy*: statements were adapted from literature review on significant knowledge of teenage pregnancy by developing 10 statements on a 3-answer namely correct, wrong, and uncertain, each statement on correct answer was scored 1, wrong or uncertain, scored 0. *Part 7 Self-care behaviors*: the last part was self-care behaviors scale including 30 items on a 4-point rating scale ranging from 0 (never) to 4 (Usually). The 25 first items on the general health care dimension, while the 5 last items on dimension of specific trimester namely first, second, and third trimesters. 5-item on each trimester were be the difference and each participant got the scale according to their current GA. Content validity was reviewed and approved by four experts in pediatric and adolescent medicine, family science, behavioral science and obstetrics & gynecology nursing. Data were verified with internal consistency reliability coefficients between 0.72 – 0.92 (Table 3).

**Table 1:** Charactersitics of participants and their husbands

Charactersitics	Frequency	Percentage
***Participants’ information***		
**Family type**		
Nuclear family	145	70.40
Extended family	61	29.60
**Age**		
14 – 15 years old	29	14.10
16 – 19 years old	177	85.90
*Range = 14 – 19, Mean±S.D.= 17.26±1.40*		
**Education level**		
Primary school	26	12.60
Secondary school	128	62.10
High school or equivalent	52	25.30
**Marital status**		
Married and living with their husband	150	72.80
Married and not living with their husband	46	22.30
Single/Divorced	10	4.90
**Work status**		
Employed	117	56.80
Unemployed	62	30.10
Student	27	13.10
**Economic status**		
Sufficient	188	91.30
Insufficient	18	8.70
***Their husbands’ information***		
**Age**		
15 – 19 years old	76	36.90
20 – 38 years old	130	63.10
*Range = 15 – 38, Mean±S.D. = 21.62±4.51*		
**Education level**		
Primary school	37	18.00
Second school	114	55.30
High school or equivalent	55	26.70
**Work status**		
Employed	128	62.20
Unemployed	58	28.10
Student	20	9.70

**Table 2:** Pregnancy information of participants

Items	Frequency	Percentage
**Current gestational age**		
First trimester (1 – 12 wks)	11	5.30
Second trimester (13 – 26 wks)	95	46.20
Third trimester (27 – 42 wks)	100	48.50
*Range = 8 – 42, Mean±S.D.= 26.36±8.54*		
**Gestational age at first ANC**		
Less than or equal to 12 weeks More than 12 weeks	97	47.10
More than 12 weeks	109	52.90
*Range = 5 – 35, Mean±S.D.= 13.97±5.44*		
**Plan for the current pregnancy**		
Yes	125	60.70
No	81	39.30
**Plan for self child rearing of their babies**		
Yes	159	77.20
No	47	22.80

**Table 3:** Characteristics of scales

Scales	Reliability coefficient	Range	Mean ± SD	%Mean^c^
Self-care behaviors (Overall SC)		63 - 118	92.30 ± 8.20	76.91
- General health care	0.77^a^	52 - 98	76.59 ± 6.50	80.06
- Specific trimester				
First trimester (n =11)	-	5 - 20	12.09 ± 3.56	60.45
Second trimester (n =95)	-	5 - 20	11.52 ± 3.60	57.58
Third trimester (n =100)	-	5 - 20	12.93 ± 3.25	64.65
Accessibility to health services (AHS)	0.72^a^	14 - 25	20.19 ± 2.54	80.76
Perceived social support from family (SSF)	0.92^a^	22 - 50	41.64 ± 5.49	83.27
Self-esteem (SE)	0.81^a^	25 - 50	41.17 ± 4.77	82.33
Knowledge on self-care during pregnancy (KN)	0.74^b^	0 - 15	10.06 ± 2.90	67.06
Perceived self-efficacy of self-care (SEF)	0.85^a^	23 - 50	40.95 ± 5.34	81.90

a Cronbach’s alpha coefficient

b KR-21reliability coefficient

c convert a mean score to a percentage

### 3.3 Statistical Analysis

The SPSS for Windows was used in both descriptive and inferential statistical analysis of the collected data. Descriptive statistics were frequency, percentage, range, mean and standard deviation. In addition, Pearson’s product moment correlation coefficient and stepwise multiple regression analysis were applied in finding the factors correlating and predicting the self-care behaviors of Thai teenagers with primigravida. Significant level was set at < 0.05

## 4. Results

The participants included 206 pregnant teenagers with an average age of 17.26 years. The average age of their husbands was 21.62 years old. Approximately 60% of the participants and their husbands had an education at secondary school level. Nearly three-fourths of the sample (70.4%) resided in a nuclear family and 72.8% were married and lived with their husband. About one-third (30%) was unemployed and more than half the samples reported that they had sufficient incomes. Concerning pregnancy information, the current GA of data collection ranged from 8-42 weeks (Mean±S.D = 26.36±8.54). Approximately, a half of them were in the third trimester and 4.5 in 10, in the second trimester. Their first ANC were at 5-35 weeks of gestation, 47.1% were at less than or equal to 12 weeks and two-thirds had planned for the current pregnancy while more than three-fourths planned for self child rearing of their babies (Table 1-2).

Considering the self-care behaviors (SC) regarding the scores of percentage-mean, they were found that the score of general health care dimension was higher than the score of SC in the specific trimester. In addition, the score of SC in the second trimester was lower than the scores in the first and third trimesters (57.58, 60.45, and 64.65, respectively). Conversely, the score of overall self-care behavior was about 77 and the score of general health care dimension was around 80. Moreover, the study showed that the percentage-mean scores of SSF, SE, SEF, and AHS were more than 80 and only KN was lower (Table 3).

By Pearson’s product moment correlation coefficient analysis, it showed that SEF, SSF, KN, SE, AHS and age were correlated with overall SC: r = 0.47, 0.34, 0.28, 0.24, 0.19 and 0.15 respectively, while coefficients between family type and GA at first ANC and SC behavior were not significant (p > .05). The six independent variables were significantly correlated with self-care behaviors. These variables were analyzed to see how it influences overall self-care behavior by using Multiple Linear Regression Analysis (MRA). The multicollinearity is a crucial assumption of MRA. Therefore, the inter-correlation coefficients among predictors were used to verify this problem. The finding showed that inter-correlation coefficients were not highly correlated (r < 0.7) (Table 4). In addition the collinearity statistic showed that the tolerance value closed up zero and VIF value, less than 10. The result of Stepwise MRA revealed that SEF (B = 0.65, beta = 0.42) and KN (B = 0.55, beta = 0.20) were both significant predictors accounted for 25% of the variance in the overall self-care behavior of Thai teenagers with primigravida (Table 5). This relationship might be shown in an equation as:

**Table 4:** Pearson’s product moment correlation coefficients (r)

Variables	Self-care	Age	Family	FANC	AHS	SSF	SE	KN
Age	0.15*							
Family type^1^	0.11	0.01						
GA at first ANC (FANC)	0.07	-0.15*	0.04					
Accessibility to health services (AHS)	0.19**	0.04	0.06	0.03				
Perceived social support from family (SSF)	0.34***	-0.01	-0.06	0.04	0.38***			
Self-esteem (SE)	0.36***	0.05	-0.05	-0.10	0.41***	0.48***		
Knowledge on self-care during pregnancy (KN)	0.28***	0.12	-0.01	0.10	0.12	0.20***	0.23***	
Perceived self-efficacy of self-care (SEF)	0.46***	0.06	0.05	0.01	0.32***	0.63***	0.50***	0.19**

1 refernece group: Nuclear family

* p-value < 0.05,

** p-value < 0.01,

*** p-value < 0.001

**Table 5:** Stepwise multiple regression analysis

Variables	B	Beta	t-value	Tolerance	VIF
Perceived self-efficacy of self-care	0.65	0.42	6.80***	0.96	1.04
Knowledge on self-care during pregnancy	0.55	0.20	3.15**	0.96	1.04
Constant	60.22		15.06***		

R^2^ = 0.25, R^2^ adjusted = 0.24, Durbin-Watson = 2.11

SC = 60.22 + 0.65 SEF + 0.55 KN

## 5. Discussion

This study was conducted in a semi-urban area of the North-Eastern region of Thailand. Most of the Thai primigravida teenagers, participants in this study, resided in a nuclear family and were married and lived with their husband. About a half of participants had their gestational age in the third trimester and GA at the first ANC was older than 12 weeks (13-35 weeks). The majority of samples had planned for current pregnancy and planned for self child rearing of their babies. According to the finding of previous studies found that the GA at first ANC of Thai primigravida teenagers was between 10 and 24 weeks. Due to the lack of planning, they started to seek ANC services later than when they should have (Chayathab, 2006; Lertsakornsiri, 2009). As regards the samples of the present study, they sought ANC services after the first trimester, which was not in compliance with the criteria for quality ANC by the strategic plan in 2010-2013 of Department of Health, Thailand (2010).

The study finding also indicated that self-care behaviors of participants were inappropriate particularly the self-care in the specific trimesters. The results were similar to the finding of a previous study by Chayathab in 2006 and Wong-Arsa and Sitkul-A-Nan in 2008, which conducted in semi-urban areas near the Bangkok metropolitan. However, this result was inconsistent with the Lertsakornsiri’s finding in 2009, which conducted with primigravida teenager in Bangkok Metropolitan area. She found that the majority of Thai participants had the score of self-care behavior at a high level or suitable self-care behaviors. One plausible explanation is that the samples were 16 to 19 years old, they were pregnant for the first time, and they completed only early secondary education. In addition, they lacked of both knowledge and experience of self-care during pregnancy. Likewise, a study conducted in the United States found that pregnant teenagers realized that they needed to eat nutritious food and exercise during pregnancy, but in practice they were unable to choose nutritious food or beneficial exercise due to the lack of knowledge of the type of food and exercise that would benefit them (Wise & Arcamone, 2011).

In this study, the factors that were found to be not associated with SC of subjects were the family type, GA at first ANC, plan for the current pregnancy and plan for self child rearing of their babies. This can be explained that most of samples had similar education levels, so they had similar ability to search for the information on self-care. Moreover, even though the samples were unemployed, they had sufficient income and most lived with older husbands who worked and had sufficient income to take care of their family. As well, most of the subjects planned for current pregnancy and had plans for self child rearing of their babies, to explain further they was a high perceived social support from their family and they were easy to accessibility to health care service (scores of percentage-mean > 80). On the contrary, this study showed that the samples sought ANC when their gestational age over 12 weeks. The result showed that most of the respondents lacked awareness about the importance of attending ANC clinics in the first trimester, this translating into their lack of knowledge on self-care. The study also found that the samples had low knowledge on self-care during pregnancy (score of percentage-mean < 70) in consequence they had inappropriate SC behaviors as well.

Furthermore, the findings indicated that SEF, SSF, KN, AHS, SE, and age were associated with SC behaviors of Thai teenagers with primigravida. This can be explained that in order for individuals to successfully practice behaviors, they need to have perceived self-efficacy to determine their own ability to manage and perform the behaviors until they accomplish their goal (Bandura, 1997). Perceived self-efficacy is a qualification of individuals who have the potential to take care of themselves, individuals who have knowledge, thinking ability, and skills to employ cognitive and intellectual processes to memorize and utilize knowledge in actual practices (Orem, 2001). In general, age is a biological variable which indicates individuals’ changes over time. It affects individuals’ levels of behaviors. Experience, learning, and training come with age, and age indicates individuals’ maturity which makes individuals express behaviors differently. Moreover, love, warmth, and care from family members are considered the support from the family which makes individuals develop encouragement and morale to perform self-care. Consequently, perceived social support from family was positively related to health care behaviors of pregnant teenagers with statistical significance (Schwartz, Vieira & Geib, 2011). Also, perceived social support from family and advice received from health care providers can reduce psychosocial problems of pregnant teenagers. Besides, self-esteem makes individuals accept and believe in themselves. It makes them feel that they are valuable and significant. Self-esteem has an influence on individuals’ positive behaviors, thus resulting in different self-care behaviors of individuals (Rosenberg, 1965). Similarly, previous studies had reported that there was a statistically significantly positive relationship between self-esteem and self-care behaviors of pregnant teenagers (Callaghan, 2006; Lertsakornsiri, 2009). Besides, in order for individuals to perform behaviors successfully, they have to have the perceived self-efficacy. They also need to have the knowledge that enables them to actually and correctly perform the behaviors. As a consequence, perceived self-efficacy and knowledge on self-care during pregnancy could predict the variation of self-care behaviors of primigravida teenagers.

Based on the study findings, there are three recommends for the related agencies and health care providers. First, antenatal care units should give the significance to the dissemination of knowledge on self-care among teenagers with primigravida, with an emphasis on the knowledge of self-care and particularly each specific trimester. The knowledge provided to these pregnant women should have enough detail to enable pregnant women to actually apply it during their pregnancy. Second, the second trimester is the period which the fetus has continuous developments and growth. However, in actual practice, the self-care during this trimester receives less significance and attention than the other trimesters. Therefore, the awareness of pregnant teenagers should be raised to enable them to continuously perform on necessary self-care. Knowledge on correct self-care practices should be emphasized in every time when they seek antenatal care services. And their past self-care behaviors should be assessed that necessary knowledge and advice on beneficial self-care activities can be given to them to ensure quality pregnancy from the beginning to the end. Finally, activities should be organized to promote self-esteem and perceived self-efficacy for self-care behaviors of primigravida teenagers. Training should be arranged in the form of group discussions among pregnant teenagers to enable them to exchange attitudes and viewpoints and develop positive feelings toward themselves. Therefore these make teenagers realize their value and develop their confidence to continuously perform appropriate self-care behaviors until their child delivery. The group leaders should be equipped with the skills to offer consultancy, persuade, and encourage pregnant teenagers to perform self-care. Moreover, groups of pregnant teenagers can be formed (such as the friends helping friends for beloved babies group) to offer them the opportunity to exchange ideas and experiences in self-care and care of the fetus so as to increase their spiritual morale and empower primigravida teenagers to continue necessary self-care for the sake of themselves and their babies. Even though both perceived self-efficacy and knowledge on self-care during pregnancy could predict the self-care behaviors of pregnant teenagers, there may be other factors which have an effect on their self-care behaviors. Some factors such as perceived health status, perceived barriers to care, perceptions of the husband or family are other important variables suggested to include in further study.
